# A cluster randomized trial assessing the impact of personalized prescribing feedback on antibiotic prescribing for uncomplicated acute cystitis to family physicians

**DOI:** 10.1371/journal.pone.0280096

**Published:** 2023-07-31

**Authors:** Greg Carney, Malcolm Maclure, David M. Patrick, Anat Fisher, Dana Stanley, Ken Bassett, Colin R. Dormuth

**Affiliations:** 1 Therapeutics Initiative, University of British Columbia, Vancouver, BC, Canada; 2 Department of Anesthesiology, Pharmacology and Therapeutics, University of British Columbia, Vancouver, BC, Canada; 3 British Columbia Centre for Disease Control, Vancouver, BC, Canada; 4 School of Population and Public Health, University of British Columbia, Vancouver, BC, Canada; 5 Department of Family Practice, University of British Columbia, Vancouver, BC, Canada; Azienda Ospedaliera Universitaria di Perugia, ITALY

## Abstract

**Objective:**

To evaluate the impact of personalized prescribing portraits on antibiotic prescribing for treating uncomplicated acute cystitis (UAC) by Family Physicians (FPs).

**Design:**

Cluster randomized control trial.

**Setting:**

The intervention was conducted in the primary care setting in the province of BC between December 2010 and February 2012.

**Participants:**

We randomized 4 833 FPs by geographic location into an Early intervention arm (n = 2 417) and a Delayed control arm (n = 2 416).

**Intervention:**

The Education for Quality Improvement in Patient Care (EQIP) program mailed to each FP in BC, a ‘portrait’ of their individual prescribing of antibiotics to women with UAC, plus therapeutic recommendations and a chart of trends in antibiotic resistance.

**Main outcome measures:**

Antibiotic prescribing preference to treat UAC.

**Results:**

Implementing exclusion criteria before and after a data system change in the Ministry of Health caused the arms to be unequal in size–intervention arm (1 026 FPs, 17 637 UAC cases); control arm (1 352 FPs, 25 566 UAC cases)–but they were well balanced by age, sex and prior rates of prescribing antibiotics for UAC. In the early intervention group probability of prescribing nitrofurantoin increased from 28% in 2010 to 38% in 2011, a difference of 9.9% (95% confidence interval [CI], 9.1% to 10.7. Ciprofloxacin decreased by 6.2% (95% CI: 5.6% to 6.9%) and TMP-SMX by 3.7% (95% CI: 3.1% to 4.2%). Among 295 FPs who completed reflective surveys, 52% said they were surprized by the *E*. *coli* resistance statistics and 57% said they planned to change their treatment of UAC.

**Conclusion:**

The EQIP intervention demonstrated that feedback of personal data to FPs on their prescribing, plus population data on antibiotic resistance, with a simple therapeutic recommendation, can significantly improve prescribing of antibiotics.

**Trial registration:** ISRCTN 16938907.

## Introduction

Indicators show that infections caused by antimicrobial resistant organisms continue to increase in Canada [1], and that while antimicrobial stewardship programs reduced the prescriptions for children, the quality of antibiotic prescribing for adults remained suboptimal [2–4]. Continued efforts to preserve the effectiveness of antimicrobials to treat infectious disease is essential since misuse of antimicrobials is the primary mechanism driving resistance [5–8]. The treatment of cystitis is one indication where a change in antibiotic use was necessary. Uncomplicated acute cystitis (UAC) is the most prevalent form of urinary tract infection in women, and is most commonly caused by *Escherichia coli* (*E*. *coli*) [9, 10]. UAC is a common indication for antimicrobial treatment in healthy, non-pregnant women [11, 12]. *E*. *coli* resistance to trimethoprim-sulfamethoxazole (TMP-SMX) and fluoroquinolones, historically the antibiotics most often used for UAC, exceeded 20% in all areas of British Columbia by 2012. Due to increased resistance, Nitrofurantoin treatment of infections caused by *E*. *coli* is now considered the treatment best supported by evidence [13].

In 2010, as part of a collaboration between the British Columbia (BC) Ministry of Health and the BC Medical Association, an initiative known as Education for Quality Improvement of Patient care (EQIP) mailed family physicians (FPs) confidential feedback portraits of their individual prescribing of antibiotics for UAC. In 2012, after the portrait was distributed, the Ministry abruptly terminated the EQIP initiative and access to the original EQIP data [14]. In 2019, under new leadership and based on a report from the BC Ombudsperson, the Ministry decided to support a re-launch of the program, and restored access to original EQIP data. The objective of this study was to evaluate the impact of personalized feedback portraits on the treatment of UAC, and to assess physician attitudes towards the EQIP program. Prior to this evaluation and based on similar audit and feedback initiatives [15, 16], we predicted the most likely impact of the portrait would be a 5–10% increase in the use of nitrofurantoin and a proportional decrease in the use of ciprofloxacin and trimethoprim-sulfamethoxazole (TMP-SMX).

## Methods

### Study design and setting

The intervention was conducted in the primary care setting in the province of BC between December 2010 and February 2012. FPs were identified through the College of Physicians and Surgeons of British Columbia registrant directory containing public information on individuals currently registered and licensed with the College. The EQIP portrait was a cluster randomized control trial. The unit of randomization was the practice community (n = 116). Pairs were matched based by size and rural vs urban location using the same method used in a prior cluster randomized control trial [17]. We assigned half of FPs (n = 2 416) based on the community where they practiced to receive the portrait with a one-year delay, who would provide the assumed counterfactual experience for the FP group who received the portrait early (n = 2 417). Prior to the portrait intervention, a registration package was sent to all family physician’s in the province announcing the EQIP program and providing them an opportunity to opt out of the initiative and its evaluation by contacting the EQIP office. The study protocol was approved by the University of British Columbia Clinical Research Ethics Board (UBC CREB number H20-00337). The ethics committee waived the requirement for informed consent. The trial is registered in ISRCTN (application 16938907) with a publicly available evaluation protocol [18]. The trial was not prospectively registered as individual recruitment was not involved and the evaluation used secondary fully anonymized administrative data.

### Randomization

Active FPs during 2009 in the province were eligible to receive the UAC portrait intervention. Physicians were included in the study if they met all of the following criteria: did not opt out of the program via their registration package; had evidence of medical practice in the form of Medical Services Plan billings in the most recent quarter of data available; were available in the BC Ministry of Health database with a valid, encrypted, and linkable MSP billing number; and were categorized as a ‘Private Practice General Practitioner” in the MSP Registration & Billing information system. Pairs of practice community locations were randomized into two groups using a random number generator. Blind to this allocation, the EQIP Working Group Chair flipped a coin to determine which group received the early intervention and which received the delayed intervention. Physicians in both groups who had zero patients diagnosed with a UAC in the prior year, resulting in an empty prescribing graph on the portrait, were sent a sample portrait with the delayed mailing, but excluded from the evaluation.

### Personalized portrait intervention

An interdisciplinary team with expertise in antimicrobial resistance, infection disease, primary care, pharmacoepidemiology, and public health developed an educational portrait aimed to improve the quality of antimicrobial prescribing for UAC in primary care. The EQIP UAC portrait (S1 File) contained individualized physician prescribing data with evidence-based messages based on *E*.*coli* resistance rates published by the BC Centre for Disease Control. The key message stated that, “Nitrofurantoin is now the first-line treatment for uncomplicated acute cystitis”. Physicians were sent a registration package several months before the portraits were mailed. The registration packages contained an information letter about the EQIP program and provided the physicians with the opportunity to opt out of the EQIP project. An article was published in the BC Medical Journal in November 2010 informing family physicians they would soon receive a confidential portrait of their prescribing of antibiotics for urinary tract infections [19]. The early intervention group received their personalized EQIP portraits by mail on December 03, 2010, and again on February 28, 2011. The delayed control group physicians were mailed their personalized EQIP portraits on February 10, 2012. The delayed control group physicians were also invited to complete a reflective activity form (S3 File) in exchange for continuing medical education credit.

The EQIP portrait was a two-page personalized colour document containing: 1) a vignette describing a common clinical encounter, 2) *E*. *coli* resistance rates for ciprofloxacin, nitrofurantoin, and TMP-SMX in BC patients which supported nitrofurantoin as first line therapy, 3) a horizontal bar graph depicting each physician’s personalized first-line prescribing for cystitis in the prior year compared with the B.C. average and the evidence based target, and 4) references and a detailed explanation of how the portrait was developed. The prescribing data was developed using prescription dispensing records, family physician visits, hospital discharge records, and patient and physician demographic information. The portraits were generated using PL/SQL Developer (Allround Automations, The Netherlands), SAS (SAS Institute, Inc., Cary, NC), and iReport Designer (Jaspersoft, Palo Alto, CA).

### Databases

Prescription records were obtained from a linked administrative database at the BC Ministry of Health. The database contains records of all prescriptions dispensed at community pharmacies with the exception of patients who were federally insured at the time (e.g., Veterans, Royal Canadian Mounted Police, and First Nations and Indigenous People). The prescription records were linked to physician services and hospitalizations using anonymous client identifiers. The physician and hospital databases contained diagnostic codes (International Classification of Disease (ICD) 9^th^ revision for physician services diagnosis, and ICD 10^th^ revision for hospital diagnosis). Data was available from April 2006 to February 2013, providing up to four years of prior medical history for the identification of patient exclusion (comorbid) conditions, and for 12 months for follow-up of portrait impact after the initial intervention mailing date.

### Prescribing endpoints

Women with visits indicating UAC were identified from MSP billing records in which the first 3 digits of the ICD-9 diagnosis code in the record indicated *595*. These UAC patients were then categorized according to whether they received antibiotic treatment on or within 3 days of the physician office visit. The primary endpoint was incident use of nitrofurantoin in the 3-day period following a diagnosis of UAC. Secondary outcomes were incident ciprofloxacin, TMP-SMX, other antibiotics, or no treatment, in the 3-day period following diagnosis. Patient visits were excluded if one of the following complicating factors were identified: abnormalities of the genitourinary tract, pregnancy, impaired renal function, spinal cord injury, multiple sclerosis, recurrent urinary tract infection, or diabetes (S2 File). In addition, FPs were asked to complete a reflective survey (S3 File) where they were asked their opinions about the accuracy of their prescribing data, information sources of bacterial resistance rates, evidence and guidelines, and if they planned to change how they treat UAC.

### Statistical analysis

We measured the percentages of women coded with UAC who received any antibiotic during the 12-month post-intervention window (Dec 3, 2010 to Dec 3, 2011) in the Early and Delayed arm, as well as the FPs’ preferences for each type of antibiotic. We compared the preferences both by calculating differences with 95% confidence intervals (CI), and by estimating preference odds ratios (OR) via logistic regressions in which the ratio of change in the intervention group between the pre-intervention period to the post-intervention period was compared to the same ratio of change (or non-change) in the delayed control group [17]. We used generalized estimating equations to calculate the preference odds ratio confidence intervals, adjusting for clustering of patients using an independent correlation structure. We included pre-intervention data to adjust for any non-random imbalances in group treatment preferences at baseline resulting from re-construction and re-execution of the algorithm for exclusions that was necessitated by the Ministry’s data system change. As a secondary analysis, we examined the duration of impact by charting the preferences in the pair of 3-month windows immediately pre and post, and the adjacent pair of 9-months windows (4–12 months pre and post).

## Results

### Physician demographics

**Fig 1:**
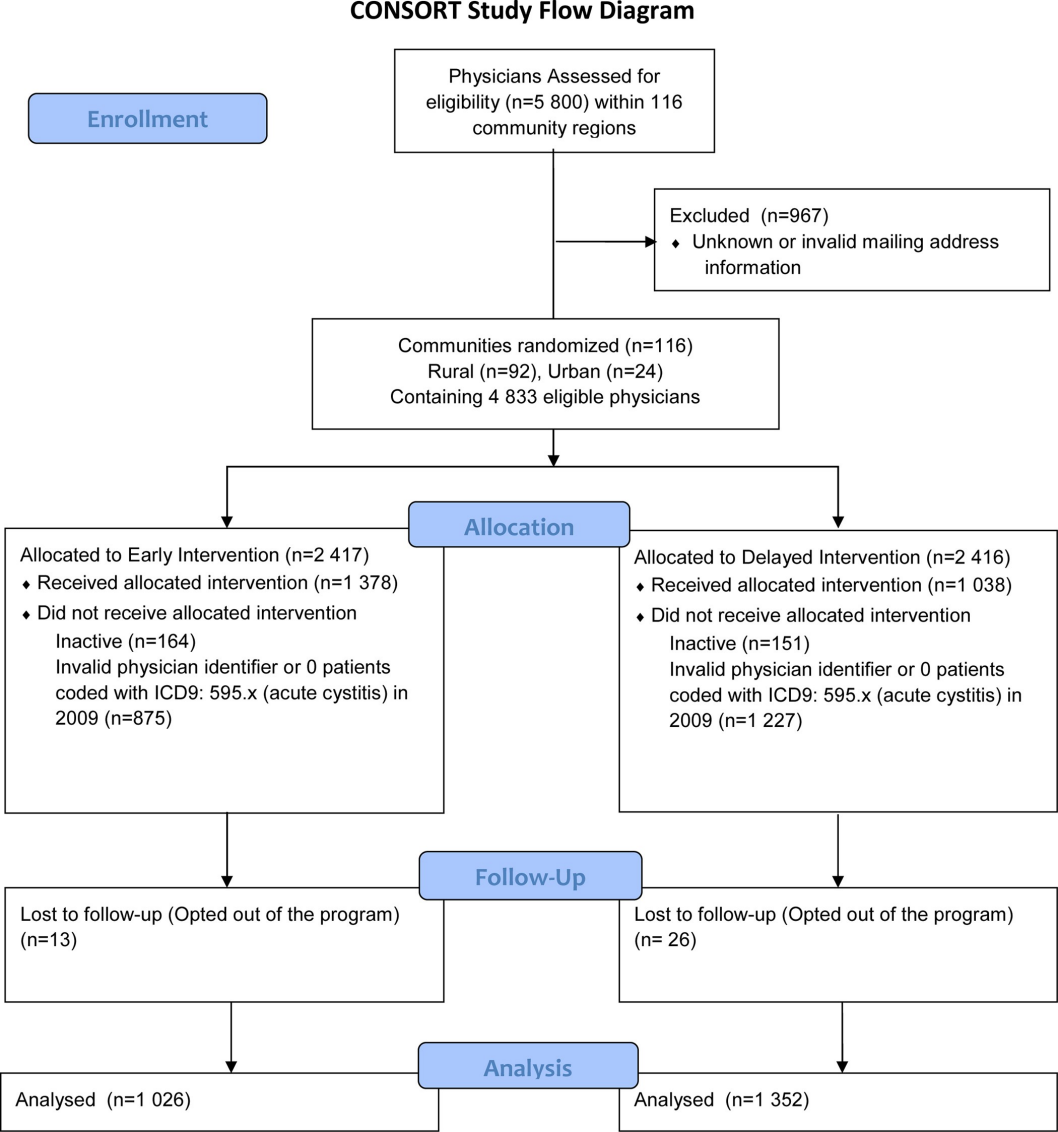
Study physician flow diagram.

The target population was 4,832 family physicians from British Columbia. 2,416 physicians did not meet the eligibility criteria, and 39 physicians opted out of the program (Fig 1). After re-construction and re-execution of the algorithm for exclusions, the early intervention arm consisted of 1,026 FPs and 17 637 UAC cases (some patients more than once), and the delayed control arm consisted of 1,352 FPs and 25 566 UAC cases. A comparison of physician characteristics between the arms is shown in Table 1. The balance by physician age and sex was better than the balance by geographic region, reflecting the fact that the unit of randomization was small geographic areas, some of which contain many FPs. Despite some geographic imbalance, the balance of preferences for the different types of antibiotics in the year before the intervention was remarkably good.

**Table 1:** Physician characteristics.

Physician Characteristics	Early Intervention Group		Delayed Control Group	
Family physicians (n)	**1026**	** **	**1352**	
Female (n, %)	340	33.1%	482	35.7%
Age as of Dec 03, 2010 (mean, IQR)	54.6	[47.4–61.5]	51.1	[43.7–58.2]
Age < 30, %	0	0.0%	4	0.3%
Age 30–39, %	48	4.7%	224	16.6%
Age 40–49, %	300	29.2%	409	30.3%
Age 50–59, %	363	35.4%	433	32.0%
Age 60–69, %	262	25.5%	245	18.1%
Age ≥ 70, %	53	5.2%	37	2.7%
Years since graduation as of Dec 03, 2010 (mean, sd)	27.5	9.8	23.7	10.7
Geographical Distribution by Health Authority				
01-Interior	143	13.9%	291	21.5%
02-Fraser	277	27.0%	327	24.2%
03-Vancouver Coastal	305	29.7%	375	27.7%
04-Island Health	247	24.1%	271	20.0%
05-Northern	47	4.6%	59	4.4%
Unknown	7	0.7%	29	2.1%
Total UAC patients, 12 months pre-intervention	23 730		33 640	
Prescriptions for pre-intervention UAC patients				
Nitrofurantoin	6 267	26.4%	8 806	26.2%
Ciprofloxacin	5 565	23.5%	7 953	23.6%
TMP-SMX	2 994	12.6%	4 609	13.7%
Other antibiotic	1 118	4.7%	1 650	4.9%
No antibiotic	7 786	32.8%	10 622	31.6%

### Primary and secondary outcomes

**Fig 2:**
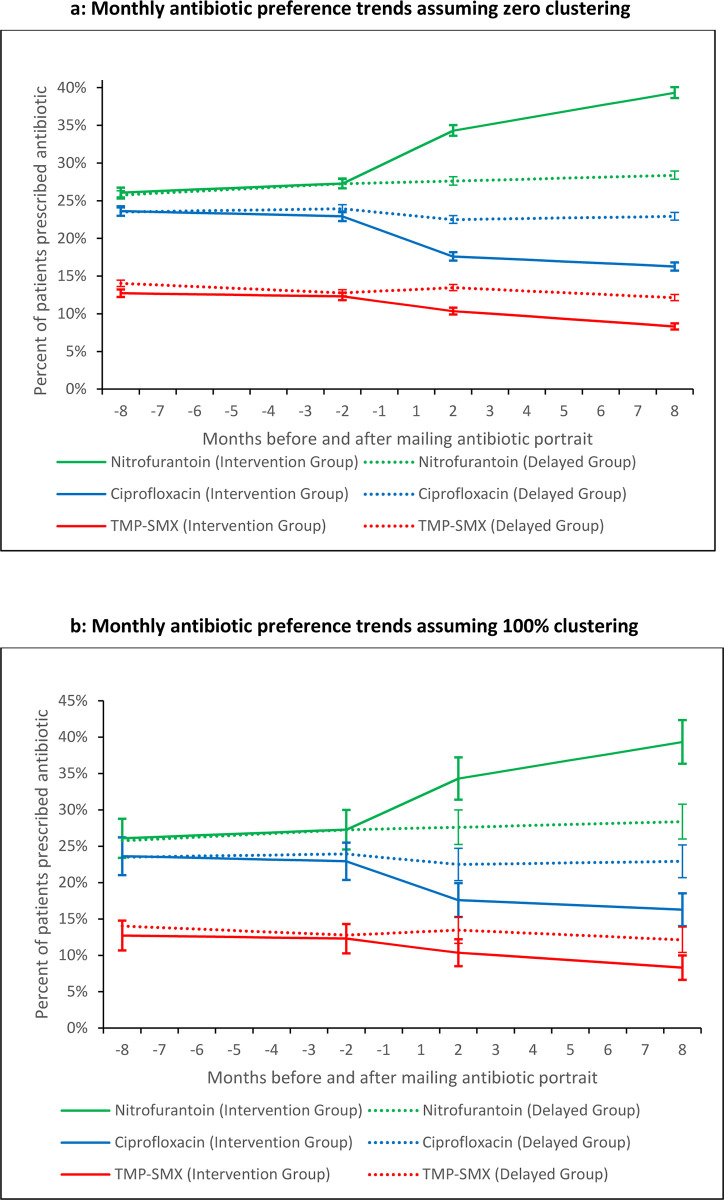
Trends in preferences. **a:** Monthly antibiotic preference trends assuming zero clustering, **b:** Monthly antibiotic preference trends assuming 100% clustering.

Tables 2 and 3 show the prescribing preference differences for UAC treatment, the mean preferences, and the preference odds ratios in the 12-month and 3-month windows pre and post intervention. Fig 2 shows the trends in preferences. Preferences in the pre-intervention windows were essentially identical in the early intervention and delayed control arms. In the post-intervention window, preferences were strikingly different. The 95% confidence intervals are mostly non-overlapping despite being very conservative: by assuming a high degree of clustering within a FP’s practice, n for sample size is set at the number of exposed FPs (1 026) rather than the number of exposed patients (17 637).

**Table 2:** Prescribing outcomes, 12 months pre and post intervention.

	Intervention Physicians				Delayed Control Physicians									
	1-Year Pre Intervention		1-Year Post Intervention		1-Year Pre Intervention		1-Year Post Intervention							
	Prescriptions	Preference	Prescriptions	Preference	Prescriptions	Preference	Prescriptions	Preference	Preference Difference	Preference Difference 95% CI		Preference Odds Ratio	Preference Odds Ratio 95% CI	
Nitrofurantoin	6267	26%	8935	38%	8806	26%	9511	28%	9.9%	9.1%	10.7%	1.57	1.43	1.71
Ciprofloxacin	5565	23%	3894	38%	7953	24%	7701	23%	-6.2%	-6.9%	-5.6%	0.67	0.52	0.83
TMP-SMX	2994	13%	2068	17%	4609	14%	4205	12%	-3.7%	-4.2%	-3.1%	0.68	0.50	0.86
Other	1118	5%	938	9%	1650	5%	1502	4%	-0.5%	-0.8%	-0.1%	0.89	0.63	1.16
No Treatment	7786	33%	7611	4%	10622	32%	10799	32%	0.4%	-0.3%	1.2%	1.02	0.92	1.12
Total Episodes	23730		23446	32%	33640		33718							

**Table 3:** Prescribing outcomes, 3 months pre and post intervention.

	Intervention Physicians				Delayed Control Physicians									
	1-Year Pre Intervention		1-Year Post Intervention		1-Year Pre Intervention		1-Year Post Intervention							
	Prescriptions	Preference	Prescriptions	Preference	Prescriptions	Preference	Prescriptions	Preference	Preference Difference	Preference Difference 95% CI		Preference Odds Ratio	Preference Odds Ratio 95% CI	
Nitrofurantoin	1730	27%	1994	34%	2500	27%	2252	28%	6.7%	5.1%	8.3%	1.37	1.20	1.54
Ciprofloxacin	1454	23%	1023	18%	2196	24%	1835	23%	-4.9%	-6.2%	-3.6%	0.74	0.56	0.91
TMP-SMX	780	12%	602	10%	1173	13%	1099	13%	-3.1%	-4.2%	-2.0%	0.74	0.54	0.94
Other	272	4%	246	4%	424	5%	384	5%	-0.5%	-1.2%	0.2%	0.89	0.59	1.20
No Treatment	2103	33%	1944	33%	2876	31%	2582	32%	1.8%	0.2%	3.4%	1.09	0.97	1.20
Total Episodes	6339		5809		9169		8152							

Logistic regression models, accounting for clustering of patients by physician, showed the preference odds for a nitrofurantoin prescription following a UAC diagnosis increased by 57% in the 12 months after the intervention (relative risk (RR) = 1.57, 95% confidence interval (CI): 1.43–1.71). The increase in nitrofurantoin preference was offset by a 33% decrease in treatment with ciprofloxacin (RR = 0.67, 95% CI: 0.52–0.83) and a 32% decrease in TMP-SMX (RR = 0.68, 95% CI: 0.50–0.86). The intervention was not associated with a change in treatment with other antibiotics or a change in UAC followed by no treatment. The 3 month pre-and-post intervention sensitivity analysis yielded similar results. The intervention was associated with a 37% increase in nitrofurantoin prescribing (RR = 1.37, 95% CI: 1.20–1.54) in the early physician group relative to the delayed group. Likewise, both ciprofloxacin and TMP-SMX were associated with a 26% decrease in treatment (RR = 0.74, 95% CI: 0.56–0.91) and (RR = 0.74, 95% CI: 0.54–0.94), respectively.

### Physician feedback

There were 295 physicians who completed and returned a reflective activity form (Table 4) in exchange for continuing medical education credit. Of the 286 physicians who reported the number of years since graduating medical school, the average was 24.8 years (standard deviation = 10.8). Physicians reported their place of practice as: 243 (82%) in a family practice office or clinic, 95 (32%) in a walk-in clinic, 60 (20%) in a long-term care facility, 53 (18%) in a teaching setting, 11 (4%) in a sessional clinic, and 60 (20%) in an “other” setting. Physicians were able to select more than one place of practice.

**Table 4:** Physician reflective survey results.

	Survey Questions	Total Responses, n = 295	
		n	%
**Do you think that your prescribing data accurately reflect your first-line prescribing for cystitis (based on MSP code 595)?**			
	Yes	222	75%
	No	64	22%
	No Response	9	3%
**Were you surprised by the rates of E. coli resistance to ciprofloxacin, TMP-SMX, and nitrofurantoin?**			
	Yes	153	52%
	No	136	46%
	No Response	7	2%
**With regards to all of the information in this portrait, please check all of the following that apply to you. Provide an explanation if you feel it is necessary.**			
	I learned something new	169	57%
	I am motivated to learn more	126	43%
	This information confirmed I did (am doing) the right thing	133	45%
	I am reassured	105	36%
	I am reminded of something I already knew	107	36%
	I am dissatisfied	4	1%
	There is a problem with this information	23	8%
	I disagree with the content of this information	4	1%
	I think this information is potentially harmful	1	0%
	This information has no impact at all on me or my practice	5	2%
**What sources do you regularly use for information on bacterial resistance and updated antimicrobial guidelines?**			
	Laboratory or hospital reports	106	36%
	British Columbia Ministry of Health or provincial Health Authority reports	56	19%
	Continuing Medical Education courses, conferences, or academic detailing	50	17%
	Medical journal articles	46	16%
	British Columbia Center for Disease Control, Do Bugs Need Drugs program	43	15%
	Uptodate.com	34	12%
	Sanford Guide	26	9%
	Colleagues	15	5%
	Other sources (BC Medical Association/Doctors of BC, eMedicine, pepid, epocrates, drug reps, pharmacists, practice guidelines)	60	20%
**Would you now plan to change your treatment of acute uncomplicated cystitis?**			
	Yes	168	57%
	No	112	38%
	No Response	15	5%
**I work in the following clinical settings (select all that apply):**			
	Sessional clinic	11	4%
	Walk-in clinic	95	32%
	Family practice in office/clinic	243	82%
	Long-term care facility	60	20%
	Teaching setting	53	18%
	Not in practice	0	0%
	Other:		
	Locum	9	3%
	Emergency Department	18	6%
	Hospital	28	9%
	Other	12	4%

With regards to all the information in the portrait, 222 (75%) agreed their prescribing data accurately reflected their first-line prescribing for cystitis. 153 (52%) reported being surprised by the rates of *E*. *coli* resistance to ciprofloxacin, TMP-SMX, and nitrofurantoin. In addition, 169 (57%) indicated they learned something new, while 133 (45%) reported the information confirmed they were doing the right thing, and 126 (43%) were motivated to learn more. In the section allowing for negative feedback, 23 (8%) physicians believed there was a problem with the information, fewer than 5 physicians reported being generally dissatisfied with the portrait content, and fewer than 5 physicians disagreed with the content of the information.

## Discussion

Our study described the impact of a personalized prescribing portrait intervention on antibiotic use for UAC in the primary care setting of BC. The prescribing impact from the UAC portrait was greater than previous portraits implemented by the EQIP initiative [17], and was comparable with similar antimicrobial audit and feedback initiatives [20]. For example, a practitioner education and feedback intervention in California achieved a 71% decrease in ciprofloxacin treatment for UAC (adjusted odds ratio = 0.29, 95% CI: 0.20–0.44) [21]. Another pharmacist-led audit and feedback project in the primary care setting in the state of Michigan was able to improve guideline-concordant prescribing for UTIs from 20% at baseline to a median of 69.2% [22]. Additionally, a Canadian pragmatic randomized trial, focused on reducing antibiotic prescriptions to adults with respiratory and urinary tract infections, demonstrated the ability to make clinically important changes in antibiotic utilization in the primary care setting using educational aids and audit and feedback [23]. Other antimicrobial stewardship interventions for UAC in emergency department settings have also been effective at increasing adherence to guidelines and reductions in fluoroquinolone therapy [24, 25].

FPs were asked to complete a reflective survey where they were asked questions pertaining to the quality of evidence, accuracy of data, and potential for changing their personal treatment decisions for patients with UAC. The responses were overwhelmingly positive, with the majority of physicians indicating they learned something new and described their individual prescribing data as accurate. Less than 2% of the physicians reported being dissatisfied with the content of the portrait. The large sample of physician responses was novel, and offered important feedback and support for the future development of personal prescribing portraits. However, the physicians responding to the survey were not a random sample, and therefore careful interpretation of the results is warranted.

Several government programs in BC have aimed at reducing antimicrobial use, including educational campaigns [26] and academic detailing [27], yet a gap between evidence-based prescribing and observed treatment practice for UAC remains. Several systematic reviews have identified key strategies for developing and implementing educational interventions to improve prescribing practices [28–30]. Feedback and comparison of clinical practices is a strategy that can be used to improve compliance with a desired change in clinical behaviour [31, 32]. Clinical performance feedback has been shown to be an effective low-cost intervention in several settings for changing prescribing behaviour [17, 33, 34]. Although systematic reviews have concluded that audit and feedback overall may provide only a small benefit, a review looking at various mailed intervention techniques reported that well-constructed mailed interventions, focusing on a single topic, and providing patient data, have the potential to evoke significant changes in physician prescribing [35]. Specifically, printed educational materials providing moderate-certainty evidence and individualized messages with a specific recommendation are more likely to improve prescribing practice [36, 37], and multifaceted interventions targeting different barriers of practice changes demonstrate more effective changes compared to a single intervention [38].

A systematic review and meta-analysis of audit and feedback interventions calculated an overall adjusted risk difference of 10% improvement in the intervention groups after adjusting for baseline difference [39]. However, audit and feedback trials are difficult to compare against each other due to the heterogeneity of the interventions, making a comparison between any individual intervention and the expected results from a meta-analysis an invalid comparison. A scoping review of 15 pharmacist-led antibiotic stewardship programs in outpatient settings in the United States found there was no consistent methodology in the implementation or outcome of interest of the stewardship programs, making the results difficult to generalize [40]. Investigators have suggested that interventions that do not build upon current methods are unlikely to contribute new generalizable findings [41]. However, the same investigators also acknowledge a lack of understanding of the key mechanisms that contribute to successful impacts in the field of audit and feedback. It is important to continue publishing detailed methods and components of individual audit and feedback interventions and their relative effectiveness. This will enable future studies to better understand the key mechanisms for successful audit and feedback interventions.

Our intervention had several strengths that may have contributed to the high magnitude of impact. First, comprehensive medication dispensing data was used to twice mail up-to-date personalized prescribing charts, with actionable evidence-based prescribing targets, and peer comparison, to over 2,000 active family physicians. The components of the intervention have been shown to be highly effective [42], and this likely led to a high level of engagement with the key messages of the portrait. Second, the ability to obtain continuing medical education credits by engaging in a short online reflective exercise is also likely to have increased physician engagement. Third, the EQIP program partnered with a well-known and highly credible organization, the BC Centre for Disease Control, and their well-known *Do Bugs Need Drugs*? program, to develop the key messages and recommended changes in antibiotic prescribing. These recommendations were complemented by clear, concise, non-controversial evidence on antibiotic resistance. A fourth reason which may explain the impact, which is not necessarily a strength of the intervention *per se*, but rather the selected topic; the prevalence of nitrofurantoin prescribing was much lower than the provincial target at the time of the intervention, allowing opportunity for significant improvement in prescribing changes.

Our study had data limitations that warrant discussion. The use of administrative health claims data is subject to data quality issues. This evaluation relies on the accuracy of diagnosis coding in the MSP database, particularly the use of the ICD-9 code 595 for cystitis. Patient visits for cystitis that were incorrectly coded would not have been included in the evaluation. Another limitation of our study was the randomization of physicians by community. This created baseline geographic imbalances between the intervention and control groups that could be related to antibiotic preference for the treatment of UAC. However, this is very unlikely to explain the magnitude of impact.

## Conclusion

Our analysis showed the educational intervention was effective at increasing use of nitrofurantoin and decreasing use of ciprofloxacin and TMP-SMX for treatment of uncomplicated acute cystitis. This intervention demonstrated that high quality educational interventions with clearly stated recommendations for action, supported by robust evidence and individualize prescribing pattern comparisons, have the potential to significantly change family physician prescribing patterns in the primary care setting. Physician feedback was overwhelmingly positive towards the quality of evidence and accuracy of data, with over half indicating they learned something new. Further educational interventions with well documented methods will assist future studies identify key factors that improve evidence-based prescribing changes.

## Supporting information

S1 Checklist CONSORT 2010 checklist of information to include when reporting a randomised trial*. (DOC)

S1 File Uncomplicated acute cystitis sample portrait. (PDF)

S2 File Cystitis complicating factors, case definitions. (PDF)

S3 File Continuing Medical Education credit reflective exercise. (PDF)

S4 File Minimum data file to support primary outcome. (XLSX)

S5 File (DOCX)
