# Supplementary material for: A cluster randomized trial assessing the impact of personalized prescribing feedback on antibiotic prescribing for uncomplicated acute cystitis to family physicians
Source: PLoS One. 2023 Jul 31;18(7):e0280096. doi: 10.1371/journal.pone.0280096 (PMC10389722; doi:10.1371/journal.pone.0280096)
Supplement: S1 File — (PDF) [file pone.0280096.s002.pdf]

How often is nitrofurantoin your first choice for UTI?

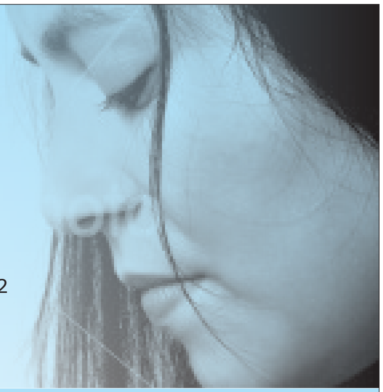

Your personal prescribing portrait for uncomplicated acute cystitis[1,2]

Clinical Vignette

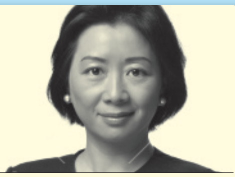

An otherwise healthy 30-year-old woman presents with frequency and dysuria. Her dipstick is positive for leukocytes and nitrites, which confirms your diagnosis of uncomplicated acute bacterial cystitis.
What would YOU prescribe?

Nitrofurantoin is now the first-line* treatment for uncomplicated acute cystitis.

Escherichia coli (E. coli) resistance to ciprofloxacin and trimethoprim-sulfamethoxazole (TMP-SMX) now exceeds 20% in BC, thus limiting the effectiveness of these treatments.

Your First-Line* Prescribing for Cystitis in 2009 with BC Average and Target BC Average First-Line Prescribing[2,3]

Prescribing data are only shown for patients coded as ICD9:**595** (cystitis)

Your Prescribing | Provincial Prescribing | Target Provincial Prescribing

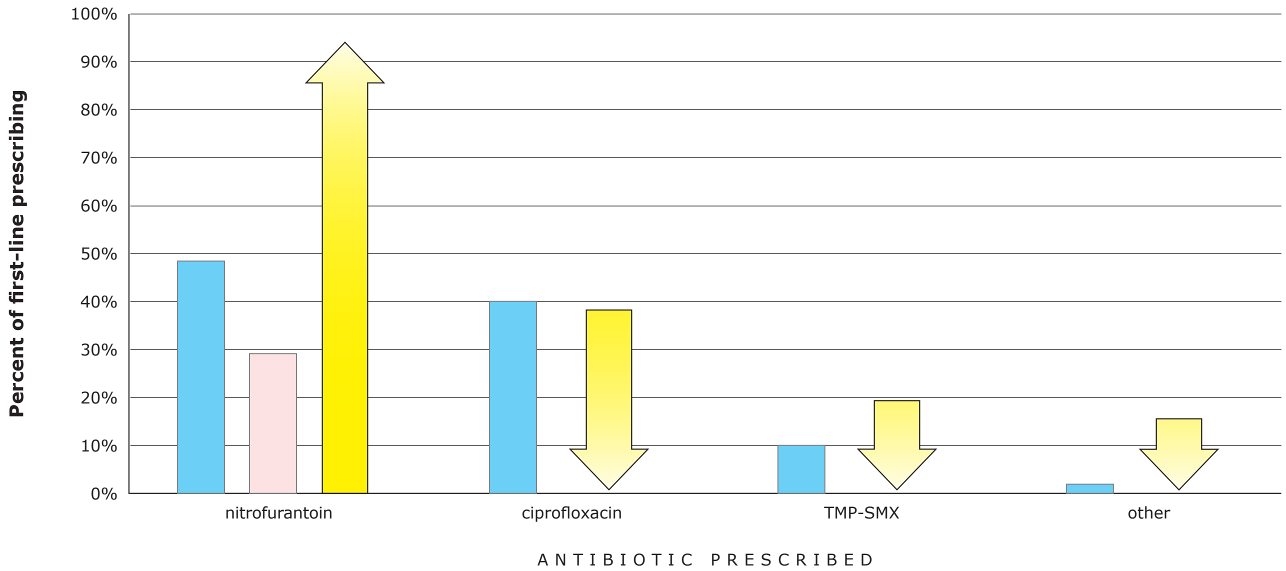

Currently recommended first-line treatment

Previously recommended first-line treatment

Not recommended first-line treatment

*First-line, in this portrait, refers to the first antibiotic dispensed within 48 hours of coding 595 in an MSP claim, i.e. generally before results of a culture.

Fluoroquinolones and trimethoprim-sulfamethoxazole are not first-line treatments for uncomplicated acute cystitis.

Rates of *E. coli* resistance to ciprofloxacin mirror the increase in fluoroquinolone utilization.

Overuse of fluoroquinolones is contributing to resistance in other enteric Gram negative organisms and can reduce the efficacy of respiratory fluoroquinolones such as moxifloxacin.

Nitrofurantoin is a narrow spectrum antibiotic that acts only on urinary pathogens. Rates of *E. coli* resistance to nitrofurantoin have remained under 5% in BC over the past 15 years, despite increasing utilization.[4]

Rates of *E. coli* Resistance to Ciprofloxacin, Nitrofurantoin and TMP-SMX[4]

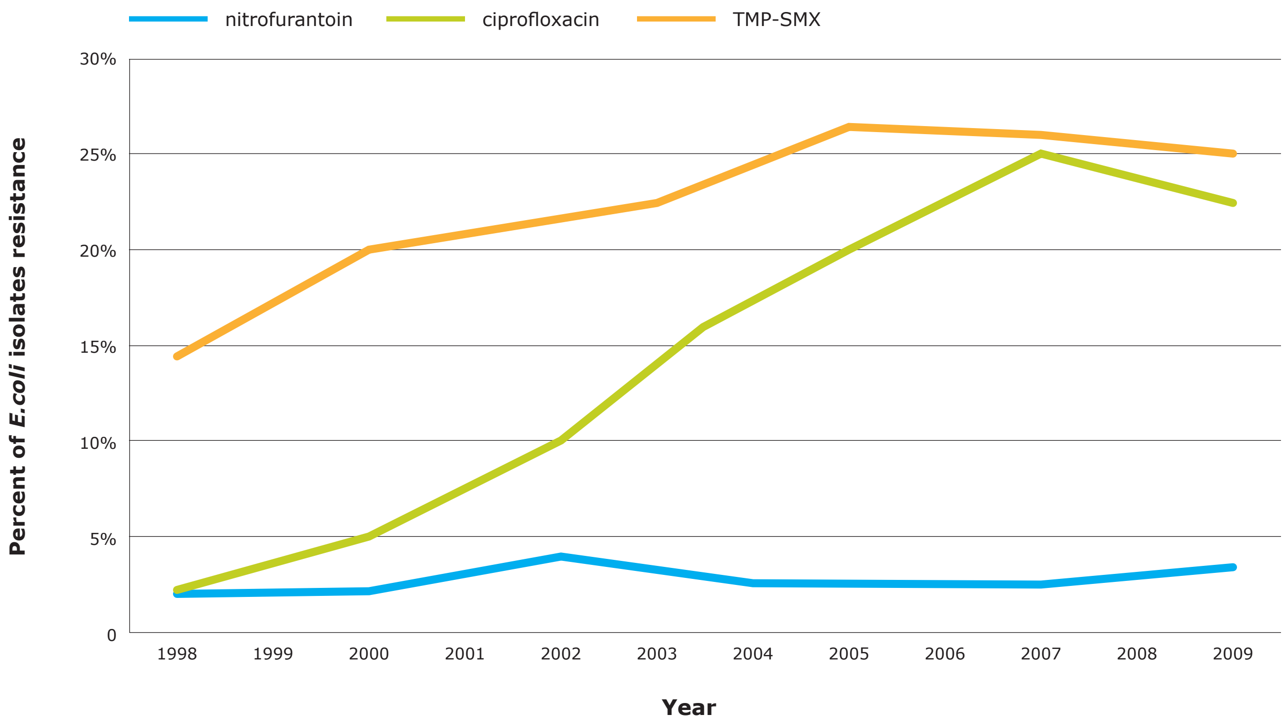

Notes

Inaccuracy in your personal prescribing portrait may arise from incomplete patient visit data or imprecise diagnosis coding.

1. **A detailed explanation of the definitions and assumptions used to create this portrait is available at www.eqip.ca**

   Messages and resistance data provided by the BC Centre for Disease Control's *Do Bugs Need Drugs?* program.

2. Where identifiable in the data, patients with complicating factors have been removed from your portrait. Approximately 25% of patients province-wide have been removed according to our criteria. Refer to www.eqip.ca for a comprehensive list of exclusions, and see the accompanying insert for guidance on treating more complicated cases.

3. "Target Provincial Prescribing" of nitrofurantoin is set at greater than 75% but less than 100% to allow for patients for whom nitrofurantoin is not indicated, such as those with an eGFR ≤40mL/min. For these patients, nitrofurantoin may not reach adequate concentration in the urine.

4. Epidemiology Services British Columbia Centre for Disease Control. Antibiotic resistance trends in the Province of British Columbia. August 2008. BC Centre for Disease Control. Available online: www.bccdc.ca/prevention/AntibioticResistance/default.htm
