# Supplementary material for: A cluster randomized trial assessing the impact of personalized prescribing feedback on antibiotic prescribing for uncomplicated acute cystitis to family physicians
Source: PLoS One. 2023 Jul 31;18(7):e0280096. doi: 10.1371/journal.pone.0280096 (PMC10389722; doi:10.1371/journal.pone.0280096)
Supplement: S2 File — (PDF) [file pone.0280096.s003.pdf]

Description	ICD 9	ICD 10	MSP fee items / Drugs	Time Period	Criteria
**Structureal, Anatomical, or Functional Abnormality of the Genitourinary Tract**					
Tuberculosis of genitourinary system	16.9	A181		April 01, 2007 to Feb 09, 2014	1 MSP claim or 1 Hospitalization*
Sequelae of genitourinary tuberculosis	137.2	B901			
Other postprocedural disorders of genitourinary system	997.5	N998			
Postprocedural disorder of genitourinary system, unspecified	997.5	N999			
Foreign body in other and multiple parts of genitourinary tract	939.3	T198			
Foreign body in genitourinary tract, part unspecified	939.9	T199			
Burn of internal genitourinary organs	947.4	T283			
Corrosion of internal genitourinary organs	947.4	T288			
Personal history of diseases of the genitourinary system	V130	Z874			
**Pregnant Females**					
Normal delivery and other indications for care in pregnancy labour and delivery	650-659.9			Jan 01, 2009 to Feb 09, 2014	1 MSP claim or 1 Hospitalization*
Complications occurring mainly in the course of labour and delivery	660-669.9			Jan 01, 2009 to Feb 09, 2014	1 MSP claim or 1 Hospitalization*
Delivery and postnatal care			14104	Jan 01, 2009 to Feb 09, 2014	1 MSP Fee Item
Delivery - attendance - emergency caesarean section			14109		
Complicated delivery - midcavity surgical delivery			4014		
Caesarean section - high risk			4025		
Caesarean section - elective			4050		
Caesarean section - emergency			4052		
Post-natal care and delivery			4104		
Caesarean section			4105		
Caesarean hysterectomy			4106		
Delivery, attendance			4109		
**Impaired Renal Function**					
Acute Glomerulonephritis	580-588.9	N00, N01, N03-N05, N07, N08, N12, N14, N17-N19		Apr 01, 2007 to Feb 09, 2014	2 MSP claims or 1 Hospitalization*
Nephrotic Syndrome					
Chronic glomerulonephritis					
Nephritis and nephropathy, not specified as acute or chronic					
Acute renal failure					
Chronic renal failure					
Renal failure, unspecified					
Renal sclerosis, unspecified					
Disorders resulting from impaired renal function					
**Spinal Cord Injury**					
Malignant neoplasm of the spinal cord	192.2	C720, C721		Apr 01, 2006 to Feb 09, 2014	2 MSP claims or 1 Hospitalization
Secondary malignant neoplasm of the brain & spinal cord	198.3	C793			
Benign neoplasm of the spinal cord	225.3	D334			
neoplasm of the brain and spinal cord	237.5	D431, D432, D434			
Diseases of the spinal cord	336.x	G320,G551,G950, G951,G952,G958-G959,G992			
Other specified anomalies of the spinal cord	742.5	Q060-Q064, Q068			
Injury to spine and spinal cord	767.4	P115			
Fracture of vertebral column with spinal cord lesion	806.x	T080, T081			
Late effect of fracture of spine & trunk	905.1	T911, T912			
Late effect of spinal cord injury	907.2	T913			
Spinal cord lesion without evidence of spinal bone injury	952.x	S140, S141.x			
Injury to nerve roots and spinal plexus	953.x	S142.x, S143.x			
**Multiple Sclerosis**					
Multiple sclerosis	340.x	G35.x		Apr 01, 2000 to Feb 09, 2014	2 MSP claims or 1 Hospitalization*
**Recurrent UTI**					
Acute Cystitis	595			Jan 01, 2007 to Feb 09, 2014	4 or more MSP visits
Other Disorders of Urethra and UT	599			Jan 01, 2007 to Feb 09, 2014	4 or more MSP visits
**Diabetics**					
