# Supplementary material for: A cluster randomized trial assessing the impact of personalized prescribing feedback on antibiotic prescribing for uncomplicated acute cystitis to family physicians
Source: PLoS One. 2023 Jul 31;18(7):e0280096. doi: 10.1371/journal.pone.0280096 (PMC10389722; doi:10.1371/journal.pone.0280096)
Supplement: S3 File — (PDF) [file pone.0280096.s004.pdf]

Complete this reflective activity for One MainPro-M1 Study Credit

Applicant Information

First name (required): ____________________ Last name (required): ____________________

CFPC member# | CFPC ID (required): ____________________

Please Note: the information above is required in order for you to receive your study credit. If you have forgotten your CFPC identification number please contact the CFPC CPD Department at **(905) 629-0900** | **1-(800) 387-6197, Ext 204.** Your National College ID Number is requested due to an arrangement between the College of Family Physicians of Canada and UBC Continuing Professional Development (UBC CPD) to provide automatic electronic transfer of continuing education credits to your college maintenance of certification profile.

In order to obtain one Mainpro-M1 study credit, please reflect on your enclosed UTI prescribing data and then complete the following form. **All your responses will be treated confidentially and only reported in aggregate with those of others.**

Your Urinary Tract Infection Prescribing Portrait for 2010

1. Do you think that your prescribing data accurately reflect your first-line prescribing for cystitis (based on MSP code 595)?

☐ **Yes** ☐ **No**

If ***Yes***, please indicate whether or not you were surprised by the results and how. If ***No***, please explain:

____

____

____

2. Were you surprised by the rates of E. coli resistance to ciprofloxacin, TMP-SMX, and nitrofurantoin?

☐ **Yes** ☐ **No**

If ***No***, where did you previously see this information?

____

____

____

3. What sources do you regularly use for information on bacterial resistance and updated antimicrobial guidelines?

____

____

____

4. With regards to all of the information in this portrait, please check all of the following that apply to you. Provide an explanation if you feel it is necessary.

- ☐ I learned something new
- ☐ I am motivated to learn more
- ☐ This information confirmed I did (am doing) the right thing
- ☐ I am reassured
- ☐ I am reminded of something I already knew
- ☐ I am dissatisfied
- ☐ There is a problem with this information
- ☐ I disagree with the content of this information
- ☐ I think this information is potentially harmful
- ☐ This information has no impact at all on me or my practice

5. What did you like, not like, or find unclear or invalid about your personal prescribing portrait?

6. Would you now plan to change your treatment of acute uncomplicated cystitis?

☐ **Yes** ☐ **No**

If ***Yes***, please explain what you plan to do differently. If ***No***, please explain why not. Please be specific and justify your answer (make an argument and/or provide references) in 2–4 sentences.

Your Demographics

1. I graduated from medical school in: _____________ (year)

2. The main region I practice in is: (select one) ☐ Rural ☐ Urban

3. I am: ☐ Female ☐ Male

4. I work in the following clinical settings: (select all that apply)

- ☐ Sessional clinic
- ☐ Walk-in clinic
- ☐ Family practice in office/clinic
- ☐ Long-term care facility
- ☐ Teaching setting
- ☐ Not in practice
- ☐ Other _____________________

Anonymous Feedback for EQIP Working Group (Very helpful but optional)

1. How could this UTI mail out be improved for a future mailing?

____

____

____

2. Which **previous** EQIP portraits do you recall seeing?

☐ Antihypertensives ☐ Statins ☐ Glucose test strips ☐ PPI ☐ URTI ☐ None

3. EQIP is considering the following topics for future portraits. How would you prioritize them (1-most relevant / 5-least relevant).

_____ Antibiotics for skin and soft tissue infections

_____ Anticoagulation in atrial fibrillation

_____ Antidepressants in pregnancy

_____ Asthma medications in children

_____ Oral contraceptives

4. Are there any other topics you would like to see as future EQIP prescribing portraits?

____

____

____

5. Please provide any additional comments:

____

____

____

Thank You!

To obtain your study credit, please fax this form to UBC CPD at (604) 875-5078 Attention: Nina Zoric.
Please be assured that your responses will be kept strictly confidential.

eqip Education for Quality Improvement in Patient Care · British Columbia Medical Association · British Columbia The Best Place on Earth · UBC CPD Learn Excel Innovate...for Life
