# Supplementary material for: A cluster randomized trial assessing the impact of personalized prescribing feedback on antibiotic prescribing for uncomplicated acute cystitis to family physicians
Source: PLoS One. 2023 Jul 31;18(7):e0280096. doi: 10.1371/journal.pone.0280096 (PMC10389722; doi:10.1371/journal.pone.0280096)
Supplement: S5 File — (DOCX) [file pone.0280096.s006.docx]

**Analytical Protocol**

**Evaluation of a Randomized Trial of Personalized Physician Prescribing Portraits for Uncomplicated Acute Cystitis**

**Authors:** Greg Carney

**Creation Date:**

**Last Updated:**

**Version:** V 1.2

Table of Contents

[Document Control 2](#_Toc391456524)

[A. Study Aim, Questions and Hypotheses 3](#_Toc391456525)

[B. Background 3](#_Toc391456526)

[C. EQIP Uncomplicated Acute Cystitis Portrait Mail-Out 5](#_Toc391456527)

[D. Evaluation Overview 5](#_Toc391456528)

[E. Study Population 6](#_Toc391456529)

[F. Outcomes 7](#_Toc391456530)

[F. Statistical Analysis Plan 9](#_Toc391456531)

[G. Outcome Tables 10](#_Toc391456532)

[Appendix Ia – Acute Cystitis Sample Portrait 13](#_Toc391456533)

[Appendix Ib: Printing Error – Portrait 15](#_Toc391456534)

[Appendix II: Antibiotic Drug List 16](#_Toc391456535)

[Appendix III – Complicating Factors for Acute Cystitis 17](#_Toc391456536)

[Appendix IV – Monthly Prescribing Trends 18](#_Toc391456537)

[Appendix V – EQIP Introduction Letter 19](#_Toc391456538)

[Appendix VI – Registration Questionnaire 20](#_Toc391456539)

[Appendix VII – CME Reflective Exercise 21](#_Toc391456540)

[Appendix VIII – Portrait FAQ 22](#_Toc391456541)

[REFERENCES 24](#_Toc391456542)

# Study Aim, Questions and Hypotheses

**Aim:** Determine the impact of personal prescribing feedback portraits on family physician prescribing of antibiotics for uncomplicated acute bacterial cystitis (UAC).

**Questions:** Primary analysis: Determine the change in antibiotic prescribing in the group of physicians who received a personal prescribing portrait for UAC

Secondary analysis: If a change in antibiotic prescribing in the intervention group is observed, measure persistence throughout the one-year post intervention period.

**Hypothesis:** The personal prescribing feedback portrait will be associated with an increase in nitrofurantoin prescribing, and a decrease in ciprofloxacin and TMP-SMX prescribing, in the treatment of UAC. No change in the proportion of patients receiving no drug therapy for a diagnosis of UAC.

# Background

The discovery of antibiotics is one of the most important advancements in modern medicine. Antibiotics are regularly used to treat infections caused by bacteria. The emergence of bacteria resistant to antibiotics is a common phenomenon. As bacterial strains increase resistance to an antibiotic, the antibiotic becomes less effective.

Incorrect or inappropriate antibiotic prescribing can lead to an increase in antibacterial-resistant bacteria. Knowledge translation initiatives are needed to reduce the misuse and overuse of antibiotics.

*Cystitis Treatment*

Cystitis is a bacterial infection of the bladder or lower urinary tract. Most cases are caused by a type of Escherichia coli (E. Coli) bacteria. Ciprofloxacin, a fluoroquinolone antibiotic, and timethoprim-sulfamethoxazole (TMP-SMX) have been used as standard drug therapy for treating Cystitis in British Columbia. According to The Do Bugs Need Drug surveillance report, E. Coli resistance to ciprofloxacin and TMP-SMX now exceeds 20% in BC, thus limiting the effectiveness of these treatments. ^[[1]](#endnote-1)^ Due to increased resistance, Nitrofurantoin is now the best evidence supported treatment of infections caused by E. Coli or Staphylococcus Saprophyticus.^[[2]](#endnote-2)^

*Physician Educational Intervention Effectiveness*

Knowledge translation initiatives have the potential to produce evidence-based changes in prescribing practices at a relatively low cost. Several systematic reviews have identified key strategies for developing and implementing educational interventions to improve prescribing practices.^iii,^^[[3]](#endnote-3),^^[[4]](#endnote-4)^

Systematic reviews have shown that educational interventions that rely on passive information transfer, including providing unsolicited medication information, tend to have a minimal effect on changing actual prescribing practice.^[[5]](#endnote-5)^ Alternatively, educational outreach as well as audit and feedback are among the most consistently effective interventions for prescribing improvement.^[[6]](#endnote-6)^ Feedback including individualized messages with a specific recommendation is more likely to improve prescribing practice.^[[7]](#endnote-7)^ Multifaceted interventions targeting different barriers of practice changes demonstrate more effective changes compared to a single intervention.^[[8]](#endnote-8)^

*Education for Quality Improvement in Patient Care (EQIP) Program*

The Education for Quality Improvement in patient Care (EQIP) program is a joint initiative of the British Columbia Ministry of Health, the BC Medical Association, and the University of British Columbia’s Therapeutics Initiative. The program focuses on improving prescribing practices that will lead to health outcome improvements for patients and result in expense reductions for the health care system. EQIP, in collaboration with the *Do Bugs Need Drugs?* program, developed a two-page personalized prescribing portrait on treating UAC.

This analysis examines the impact of the EQIP portrait on prescribing of nitrofurantoin, ciprofloxacin, TMP-SMX, and other antibiotics for the treatment of UAC. The analysis methods specified in this protocol builds upon the concepts previously described by Maclure, 2005^[[9]](#endnote-9)^ and Dormuth 2012.^[[10]](#endnote-10)^

# EQIP Uncomplicated Acute Cystitis Portrait Mail-Out

The portraits were developed using de-identified data provided by the British Columbia Ministry of Health. The data consisted of PharmaNet’s ClaimsHist prescription claims, Medical Services Plan physician visits, Discharge Abstract Database hospitalization records, and patient and physician demographic information. The portraits were generated using PL/SQL, SAS, and Jaspersoft’s iReport software.

Physicians were sent a registration package several months before the portraits were mailed. The registration packages contained an information letter about the EQIP program and provided the physicians with the opportunity to opt out of EQIP or agree to a paid follow-up interview.

The intervention group consisted of 1,026 family physicians. Their individualized portraits were initially mailed on December 03, 2010. However, due to a printing error that caused some distortion in the antibiotic prescribing graph (Appendix 1b) a corrected portrait was re-mailed on February 28^th^, 2011 (Appendix 1a).

There were 2,345 physicians in the control group (delayed mailing). Their individualized portraits were mailed on February 10, 2012.

# Evaluation Overview

A paired community design was used. Communities were paired according to the number of physicians and geographic similarity. The study population included family physicians from British Columbia who prescribed drug therapy for patients with UAC. The intervention was a personalized prescribing portrait on antibiotic prescribing in British Columbia that included evidence-based messaging supporting nitrofurantoin as first-line therapy.

The UAC portrait was the fourth EQIP portrait developed. Physicians who had not previously received an EQIP intervention received a registration package containing the following materials:

- Letter introducing the EQIP program with URL links for more information and contract information for the EQIP office (Appendix V).
- Registration Questionnaire with options to participate in a paid interview or to decline future EQIP mailings (Appendix VI).

The UAC intervention package contained:

- Personalized prescribing portrait on antibiotic prescribing for patients diagnosed with UAC (Appendix Ia)
- Reflective exercise to qualify for One MainPro-M1 Continuing Medical Education credit (Appendix VII)
- Urinary Tract Infection (UTI) Frequently Asked Questions (Appendix VIII)

We extract administrative health claim records for eligible patients from the source population with a diagnosis of UAC and look forward up to two days to determine antibiotic treatment type (or no treatment).

The evaluation design is a 4-way comparison of numerators. The ratio of change in the intervention group between the pre-intervention period to the post-intervention period is compared to the saem ratio of change (or non-change) in the delayed control group.

# Study Population­

- 1. **Participating Physicians**:

All active family physicians in the province were eligible to receive the EQIP UAC Portrait. Physicians were randomized into the intervention or control group if they met all of the following criteria:

1. Did not opt out of the program via their registration package
2. Were not retired, defined as having Medical Services Plan billings in the most recent quarter of data available.
3. Had a valid encrypted MSP Billing Number in both the Medical Services Plan Registration & Billing information system and the Health*ideas* practitioner roster.
4. Categorized as a ‘Private Practice General Practitioner’ in the Medical Services Plan Registration & Billing information system.

The early intervention group consists of 1,026 family physicians who received a portrait (with a printing error) on December 03, 2010, and a corrected portrait on February 28, 2011.

The delay control group consists of the 2,345 family physicians who received a portrait on February 10, 2012.

- 1. **Cystitis Patient Episodes**

UAC is defined using Medical Services Plan claims for participating family physicians who met the criteria in section E.1. UAC episodes are identified using the following steps:

1. Include female patients only.
2. Extract Medical Service Plan claims where the first 3 digits of the ICD-9 diagnosis code are 595.
3. Exclude reversed medical services billing claims from above.
4. Exclude episodes that meet the “complicating” factors as described in Appendix III – UTI Complicating Factors for Acute Cystitis
   1. **Study Period**

Due to the printing error and subsequent corrected mailing, two possible exposure periods are defined for the primary analysis:

1. December 03, 2010 to February 28, 2011
2. February 28, 2011 to February 09, 2012
3. February 10, 2012 to February 09, 2014
4. A time series analysis will look at antibiotic prescribing and UTI diagnostic coding from January 01, 2001 to Dec 31, 2019
   1. **Eligible patients**

Female patients who visited a family physician during the study period, or one-year prior to the study period, where the primary diagnosis code on the Medical Services Plan billing record was coded as 595.x (Cystitis). Reversed billing claims are excluded.

# Outcomes

The study outcome is a filled prescription for an antibiotic in one of the following categories:

- 1. **Antibiotic Groupings**

Antibiotic chemicals are grouped into the four categories (See Appendix II – Antibiotics DIN List for drug grouping details):

- - 1. Nitrofurantoin
    2. Ciprofloxacin
    3. TMP-SMX
    4. Other

No drug treatment will also be included as an outcome.

- 1. **Linking Treatment to Diagnosis**

Treated UAC defined as a prescription fill for an antibiotic, from F.1 above, within 2 days of a physician visit (prescription fill date minus Physician visit date must be between 0 and 2, inclusive) the meets the eligibility criteria of a UAC episode as describing in Section E.2

- 1. **Follow-up Time**

Patients are followed from the day they are diagnosed with a UAC episode until two days after their diagnosis, for a 3 day total follow-up window to determine if they received antibiotic treatment.

Date range for Claims History drug dispensing (all patients who filled a prescription at a community pharmacy) data to identify antibiotic prescribing in the follow-up window is from December 04, 2009 to February 09, 2012.

- 1. **Adjustment for past prescribing**

Adjust for chance imbalances in group preferences at baseline by adjusting the odds ratio for past prescribing (See Outcome Tables 2a and 2b); the pre-intervention period will be the 12 months prior to the study period start date.

- 1. **Outcome Tables**

Outcome tables 2a and 2b calculate the change in overall physician prescribing preference by comparing their ‘before’ preference to their ‘after’ preference. The before period is the 12 month period prior to each of the two study period start dates (December 03, 2010 and February 28, 2011)

Odds Ratios are adjusted for:

- Intervention period (=1 for year after, =0 for year before)
- Physician group (=1 for early intervention group, =0 for delay group)

*Estimate adjusted odds ratios using logistic regression (proc logistic)

* Delete records where Physician has unknown or invalid sex

# Statistical Analysis Plan

1. Assemble the physician cohorts and complete baseline characteristics table 1a. Calculate standardized differences to identify imbalances.
2. Identify eligible patients and their UAC episodes

2b) exclude UAC episodes that meet the criteria for complicating factors in Appendix-III

1. Complete baseline characteristics table 1b. Calculate standardized differences to identify imbalances
2. Extract antibiotic prescription records for eligible patients.
3. Apply the prescription to diagnosis linkage as described in F.2.
4. Sum group prescribing by drug, by month for each physician group. Create figures 1a-1d shown in Appendix IV – Monthly trend
5. Complete the Outcomes Tables 2a and 2b for the two study periods.
6. Calculate Odds Ratio, adjusting for factors listed in F.5.

# Outcome Tables

Notes:

1) Twelve months prior to initial mail-out date (Dec 03, 2010)

2) Twenty-four months prior to initial mail-out date

3) Twenty-four months prior to initial mail-out date

# Appendix II: Antibiotic Drug List

# Appendix III – Complicating Factors for Acute Cystitis

* ICD 10 codes are evaluated in all 25 diagnosis fields on hospital discharge records

# Appendix IV – Monthly Prescribing Trends


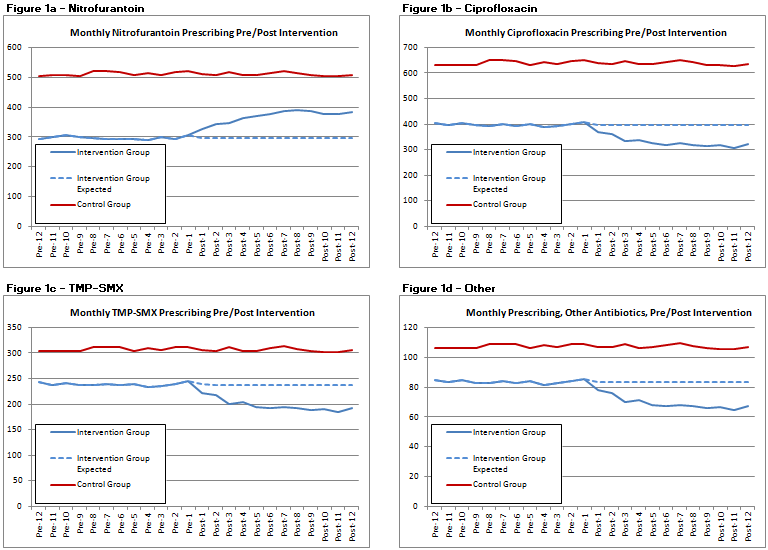


# Appendix VI – Registration Questionnaire


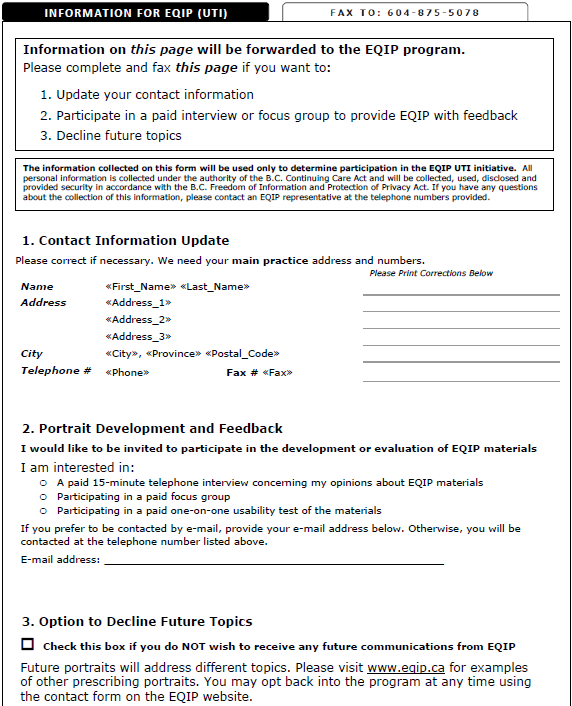


# Appendix VII – CME Reflective Exercise

# Appendix VIII – Portrait FAQ

# REFERENCES

1. BC Centre for Disease Control. Antimicrobial Resistance Trends in the Province of British Columbia, 2012. Prepared for the Do Bugs Need Drugs? Program. August 2013. [↑](#endnote-ref-1)
2. Gupta K. et al. Short-Course Nitrofurantoin for the Treatment of Acute Uncomplicated Cystitis in Women. Arch Intern Med. 2007;167(20):2207-2212. [↑](#endnote-ref-2)
3. Majumdar SR, Soumerai SB. Why most interventions to improve physician prescribing do not seem to work. CMAJ 2003; 169(1):30-31. [↑](#endnote-ref-3)
4. Ostini R, Hegney D, Jackson C, Williamson M, Mackson JM, Gurman K et al. Systematic review of interventions to improve prescribing. *Ann Pharmacother* 2009; 43(3):502-513. [↑](#endnote-ref-4)
5. Thomson O'Brien MA, Oxman AD, Davis DA, Haynes RB, Freemantle N, Harvey EL. Educational outreach visits: effects on professional practice and health care outcomes. *Cochrane Database Syst Rev*. 2000;(2):CD000409. [↑](#endnote-ref-5)
6. Thomson O'Brien MA, Oxman AD, Davis DA, Haynes RB, Freemantle N, Harvey EL. Audit and feedback versus alternative strategies: effects on professional practice and health care outcomes. *Cochrane Database Syst Rev*. 2000;(2):CD000260. [↑](#endnote-ref-6)
7. Avorn J, Soumerai SB. Improving drug-therapy decisions through educational outreach. A randomized controlled trial of academically based "detailing". *N Engl J Med* 1983; 308(24):1457-1463. [↑](#endnote-ref-7)
8. Grimshaw JM, Shirran L, Thomas R, Mowatt G, Fraser C, Bero L et al. Changing provider behavior: an overview of systematic reviews of interventions. *Med Care* 2001; 39(8 Suppl 2):II2-45. [↑](#endnote-ref-8)
9. Maclure M, Nguyen A, Carney G, Dormuth C, Roelants H, Ho K, Schneeweiss S. Measuring Prescribing Improvements in Pragmatic Trials of Educational Tools for General Practitioners. *Pharmacology & Toxicology* 2006, 98, 243–252. [↑](#endnote-ref-9)
10. Dormuth CR, Carney G, Taylor S, Bassett K, Maclure M. A Randomized trial assessing the impact of a personal printed feedback portrait on statin prescribing in primary care. *J Contin Educ Health Prof.* 2012 Jun;32(3):153-62. [↑](#endnote-ref-10)
